# Is the Expression of the Components of the Carotid Matrix of Rats Influenced by Estrogen, Progestin and Tibolone?

**DOI:** 10.1055/s-0039-1693681

**Published:** 2019-07

**Authors:** Marcelo Luis Steiner, Thérèse Rachell Theodoro, Shirley Gimenez Garcia, Ana Maria Amaral Antonio Mader, Luciano de Melo Pompei, Maria Aparecida da Silva Pinhal, César Eduardo Fernandes

**Affiliations:** 1 Department of Gynecology and Obstetrics, Faculdade de Medicina do ABC, Santo André, SP, Brazil; 2 Department of Biochemistry, Faculdade de Medicina do ABC, Santo André, SP, Brazil; 3 Faculdade de Medicina do ABC, Santo André, SP, Brazil; 4 Department of Pathology, Faculdade de Medicina do ABC, Santo André, SP, Brazil

**Keywords:** extracellular matrix, proteoglycans, atherosclerosis, estrogen, progestogen, tibolone, matriz extracelular, proteoglicanos, aterosclerose, estrogênio, progestogênio, tibolona

## Abstract

**Objective** To analyze the effects of estrogen alone or in combination with progestogens and tibolone (TIB) on the expression of the extracellular matrix metalloproteinases 2 and 9 (MMP-2 and MMP-9), of perlecan, and of heparanase (HPSE) of the vascular walls of the carotid arteries.

**Methods** A total of 30 250-day-old ovariectomized Wistar rats were orally treated for 5 weeks with: a) 1 mg/kg of estradiol benzoate (EB); b) EB + 0.2 mg/kg of medroxyprogesterone acetate (MPA); c) EB + 0.2mg/kg of norethisterone acetate (NETA); d) EB + 2 mg/kg of dydrogesterone (DI); e) 1 mg/kg of TIB; f) placebo (CTR). Following treatment, the expression of mRNA for MMP-2, MMP-9, and HPSE was analyzed by real-time polymerase chain-reaction (PCR), and the expression of MMP-2, of MMP-9, of tissue inhibitor of metalloproteinase 2 (TIMP-2), and of perlecan was quantified by immunohistochemistry in the carotid arteries.

**Results** The groups showed significant differences on mRNA HPSE expression (*p* = 0.048), which was higher in the EB, EB + MPA, and TIB groups. There was no statistically significant difference in mRNA MMP-2 or MMP-9 expression. The immunohistochemical expression of MMP-2, of TIMP-2, of MMP-9, of HPSE, and of perlecan showed no differences between groups.

**Conclusion** Estradiol alone or associated with MPA and TIB treatment can increase mRNA HSPE expression of the walls of the carotid arteries in ovariectomized rats.

## Abstract (pt)

**Resumo:**

**Objetivo** Analisar os efeitos do estrogênio isolado ou em combinação com progestogênios e tibolona (TIB) na expressão das metaloproteinases 2 e 9 da matriz extracelular (MMP-2 e MMP-9), da perlecan e da heparanase (HPSE) das paredes vasculares das artérias carótidas.

**Métodos** Trinta ratas Wistar ovariectomizadas com 250 dias de idade foram tratadas oralmente por 5 semanas com: a) 1 mg/kg de benzoato de estradiol (EB); b) EB + 0,2 mg/kg de acetato de medroxiprogesterona (MPA); c) EB + 0,2mg/kg de acetato de noretisterona (NETA); d) EB + 2 mg/kg de didrogesterona (DI); e) 1 mg/kg de TIB; f) placebo (CTR). Após o tratamento, a expressão de mRNA para MMP-2, MMP-9, e HPSE foi analisada por reação em cadeia da polimerase (RCP) em tempo real, e a expressão de MMP-2, MMP-9, inibidor tecidual de metaloproteinase 2 (TIMP-2), e de perlecan foi quantificado por imunohistoquímica em artérias carótidas.

**Resultados** Os grupos apresentaram diferenças significativas na expressão do mRNA HPSE (*p* = 0,048), sendo maiores nos grupos EB, EB + MPA e TIB. Não houve diferença estatisticamente significativa nas expressões de mRNA MMP-2 ou MMP-9. A expressão imunohistoquímica de MMP-2, TIMP-2, MMP-9, HPSE e perlecan não mostrou diferenças entre os grupos.

**Conclusão** O estradiol isolado ou associado ao tratamento com MPA e TIB pode aumentar a expressão de mRNA HSPE nas paredes das artérias carótidas em ratas ovariectomizadas.

## Introduction

Cardiovascular diseases (CVDs) are the main cause of death in postmenopausal women. Evidence show that hypoestrogenism is directly involved in the risk of CDV, mainly of coronary heart disease.^1^ ^2^ The impact of estrogen therapy on cardiovascular risk is related to the “optimal window opportunity,” in which starting the therapy in the first years of menopause is thought to be beneficial. This effect is probably related to the ability of estrogen to stimulate the expression of extracellular matrix proteases (MMPs).^3^ The extracellular MMPs belong to the endoproteinase family and have the function of remodeling the extracellular matrix in physiological as well as in pathological processes.^4^ ^5^ They maintain the homeostasis of the extracellular matrix through regulated degradation and deposition of its components, which contribute toward vascular remodeling. The increase in extracellular matrix degradation by MMPs in atheromatous plaques can explain the instability of the plaque and the consequent cardiovascular event. Metalloproteinases 2 and 9 (MMP-2 and MMP-9) are strongly associated with atherosclerotic plaque instability.^6^ Perlecan is another important extracellular matrix component belonging to the proteoglycan family, which plays an important role by interacting with growth factors, such as fibroblast growth factors (FGFs), platelet-derived growth factor (PDGF), vascular endothelial growth factors (VEGFs), *inter alia*, and controlling signaling thereof.^7^ Perlecan contains a domain which is a receptor for low-density lipoprotein (LDL),^8^ and its ability to retain LDL in the extracellular matrix can be related to the development of atherosclerotic plaque.^9^ Lastly, heparanase (HPSE) is another extracellular matrix enzyme, which degrades heparan sulfate molecules and has procoagulant activity, which can be increased by estrogen.^10^ The involvement of metalloproteinases and of other extracellular MMPs in atherosclerotic disease and atheromatous plaque instability incited research to correlate their function with hormone therapy. A previous study by our group revealed that estrogen therapy is associated with higher expression of MMP-2, of MMP-9, of and perlecan in the walls of carotid arteries.^11^ Therefore, there was interest in identifying the effect of different combinations of progestogens with estrogen therapy, and also the effects of tibolone (TIB) therapy. The aim of the present study was to evaluate the effects of estrogen and progestogens, including TIB, on the expression of extracellular MMPs and proteases of the carotid arteries.

## Methods

All of the protocol procedures were approved by the animal research ethics committee of the Faculdade de Medicina do ABC, Santo André, state of São Paulo, Brazil. In this animal experimental study, a total of 30 250-day old Wistar rats were randomly selected and subjected to bilateral oophorectomy, under anesthesia with ketamine and xylazine injected intraperitoneally. The ventral longitudinal abdominal approach was used for identifying and ligating the ovarian pedicles and then removing the gonads. Three weeks after the surgical procedure, a microscopical analysis of vaginal smears of all of the rats was performed to confirm hypoestrogenism. Subsequently, the animals were randomly divided into 6 groups of 5 animals each, and the groups received one of the following treatments administered daily by gavage for 5 consecutive weeks: EB group: estradiol benzoate (EB) administered orally in a dose of 1 mg/kg; EB/MPA group: EB 1 mg/kg combined with medroxyprogesterone acetate (MPA) 0.2 mg/kg; EB/NETA group: EB 1 mg/kg combined with norethisterone acetate (NETA) 0.2 mg/kg; EB/DI group: EB 1 mg/kg combined with dydrogesterone (DI) 2 mg/kg; TIB group: TIB 1 mg/kg; CTR group: oral placebo. The animals were kept in a calm environment with a constant temperature of 23°C, a 12 hour light period per day, and water and food *ad libitum*. At the end of the treatment period, the rats were euthanized in a CO_2_ chamber. The right and left carotid arteries were immediately dissected and resected. The right carotid arteries were placed in containers with RNA-preserving reagent, RNAholder (RNAlater^TM^, Ambion, Inc., Austin, Texas, USA), and stored at - 20°C until total RNA was extracted from each sample. The mRNA complementary strand (cDNA) was obtained, and the genes for heparanase (HPSE), MMP-2 and MMP-9 were amplified. The left carotid arteries were fixed in 10% buffered formalin for subsequent immunohistochemical analysis of the protein expression of HPSE, MMP-2, tissue inhibitor of metalloproteinase 2 (TIMP-2), MMP-9, and perlecan.

### Total RNA Extraction from the Tissues, Obtaining cDNA from the Total RNA, and Quantitative Real-time Polymerase Chain Reaction

The detailed procedures for extracting total RNA from the tissues, obtaining cDNA from the total RNA, and real-time polymerase chain reaction (PCR) have been previously published.^11^ Briefly, total RNA was extracted after the carotid artery tissue had been homogenized in liquid nitrogen by adding the TRIzol reagent (Thermo Fisher Scientific, Waltham, MA, USA) and following the protocol of the manufacturer. Total RNA in each sample was quantified using a GeneQuant RNA/DNA Calculator (GE Healthcare, Chicago, IL, USA). Following total RNA extraction, the cDNA was obtained using the RT-PCR Super Script II kit (Invitrogen, Carlsbad, CA, USA) and following the protocol of the manufacturer. The cDNA obtained was stored in a freezer at - 20°C until it was used in the real-time PCR reactions. The real-time PCR was performed using cDNA (1 µg/µL) from each sample to quantify the metalloproteinase isoforms and HPSE in the tissue using primers and the ribosomal glyceraldehyde-3-phosphate dehydrogenase (GAPDH) as a reference gene for the reaction. For real-time PCR amplification, the SYBR Green reagent (Thermo Fisher Scientific, Waltham, MA, USA) and a 7500 real-time PCR System (Applied Biosystems, Foster City, CA, USA) were used, and the amplification curves were analyzed using the software that comes with the system. Since the expression of the enzymes is relative, the calculation was based on the number of amplification cycles (Cts), and the final result was obtained in relation to the average Cts of the reference gene. All of the mRNA quantifications were performed in triplicate for each sample, and the average of the triplicate was used as the final value in the analysis.

### Immunohistochemistry

The detailed procedures for the immunohistochemical analysis have been previously published.^11^ Briefly, the immunohistochemistry was performed to analyze protein immunolabeling for metalloproteinases, perlecan, and HPSE in 3 µm-thick histological slices. The primary antibodies used were anti-MMP-2 8B4 (sc-13595), anti-MMP-9 C-120 (sc-6840), anti-TIMP-2 H-140 (sc-5539), anti-perlecan H-300 (sc-25848), and anti-HPSE: HPA1 H-80 (sc-25825) (Santa Cruz Biotechnology, Inc., Dallas, TX, USA) diluted in bovine serum albumin (BSA) in a ratio of 1:300. The slides were analyzed using a Nikon Eclipse TS100 optic microscope (Nikon Instruments, Melville, NY, USA) using the same light intensity and condenser height for all slides. The areas that best represented the immunolabeling of the slide were chosen and analyzed at a 400x magnification. Photomicrographs (640 × 480 pixels) of consecutive, nonoverlapping fields were obtained using a Nikon Coolpix 4300 (Nikon Corporation, Tokyo, Japan) digital camera with maximum optical zoom. Immunohistochemical labeling was quantified using the Scion ImageLab for Windows software (Scion Corporation, Frederick, MD, USA).

### Statistical Analysis

The data obtained was organized in electronic spreadsheets using the Microsoft Excel 2007 software (Microsoft Corporation, Redmond, WA, USA). The statistical analysis was performed using the software WinSTAT, version 2007.1 (R. Fitch Software, Cambridge, MA, USA). Continuous numerical data are presented in the average ± standard deviation (SD) format. Normal distribution was tested using the Kolmogorov-Smirnov test. Group comparisons were performed by analysis of variance (ANOVA), and multiple comparisons were corrected using the least significant difference method. The Kruskal-Wallis test was performed for data without a normal distribution, or when homogeneity of variance had not been proven. A significance level of 5% was adopted.

## Results

A rat from the TIB group was found dead one morning showing signs of having been attacked by the other rats in the group. Therefore, information from 29 animals was analyzed. Table 1 shows the results for the relative quantification of mRNA for HPSE (HPSE_[RNA]_), MMP-2 (MMP-2_[RNA]_), and MMP-9 (MMP-9_[RNA]_) for each group. A statistically significant difference can be noted on HPSE mRNA expression, with higher values in the EB, EB/MPA and TIB groups. Although there is no statistically significant difference, the TIB and CTR groups showed higher levels of MMP-2_[RNA]_ and MMP-9_[RNA]_ expression.

**Table 1:** Results of the relative levels of mRNA encoding heparanase, MMP-2 and MMP-9 according to the treatment group

	EB	EB/MPA	EB/NETA	EB/DI	TIB	CTR	*p-value*
HPSE_[RNA]_	7.05 ± 5.50	5.56 ± 7.54	0.10 ± 0.16	0.64 ± 1.10	9.41 ± 18.6	1.26 ± 0.90	0.048*
MMP-2_[RNA]_	0.88 ± 0.46	0.98 ± 1.44	0.31 ± 0.25	0.30 ± 0.14	1.43 ± 2.45	1.83 ± 1.38	0.680
MMP-9_[RNA]_	0.09 ± 0.11	0.42 ± 0.78	0.19 ± 0.08	0.23 ± 0.23	1.93 ± 3.57	1.68 ± 1.56	0.433

* Kruskal-Wallis test and analysis of variance were used for the other parameters.

Abbreviations: CTR, control group; EB,estradiol benzoate group ; EB/DI, estradiol benzoate and dydrogesterone group; EB/MPA, estradiol benzoate and medroxyprogesterone acetate group; EB/NETA, estradiol benzoate and norethisterone acetate group; HPSE, heparanase; TIB, tibolone.

Regarding the immunohistochemistry results, as shown in Table 2, there was no statistically significant difference among the groups for any evaluated parameters. It is interesting to note, however, that the CTR group had lower median levels for all extracellular matrix components analyzed when compared with the other groups.

**Table 2:** Results of the immunohistochemistry quantification of HPSE, MMP-2, TIMP-2, MMP-9 and perlecan according to treatment group

	EB	EB/MPA	EB/NETA	EB/DI	TIB	CTR	*p* Value
HPSE_[IH]_	66.5 ± 28.4	67.2 ± 9.6	92.1 ± 21.0	62.1 ± 38.2	60.0 ± 25.2	57.8 ± 14.0	0.417*
MMP-2_[IH]_	36.8 ± 18.7	19.7 ± 8.3	39.0 ± 9.4	34.1 ± 18.8	30.9 ± 23.9	17.2 ± 17.1	0.382
TIMP-2_[IH]_	44.1 ± 29.1	28.6 ± 22.8	35.6 ± 13.1	34.3 ± 26.8	42.1 ± 22.7	13.5 ± 11.1	0.441
MMP-9_[IH]_	55.1 ± 12.2	48.2 ± 17.0	61.8 ± 5.9	41.8 ± 20.9	55.8 ± 25.1	44.8 ± 8.0	0.548
Perlecan	57.1 ± 11.6	36.9 ± 17.9	56.8 ± 9.8	60.1 ± 19.3	51.0 ± 6.0	50.0 ± 12.6	0.262

Abbreviations: CTR, control group; EB,estradiol benzoate group ; EB/DI, estradiol benzoate and dydrogesterone group; EB/MPA, estradiol benzoate and medroxyprogesterone acetate group; EB/NETA, estradiol benzoate and norethisterone acetate group; TIB, tibolone.

* Kruskal-Wallis test and analysis of variance were used for the other parameters.

## Discussion

As far as we know, this is the first study that endeavored to compare the greatest number of estroprogestative combinations in terms of expression of proteases of the extracellular matrix of the vascular walls. The results show a difference among the treated groups only for HPSE mRNA expression.

The median values of MMP-2 and MMP-9 mRNA expression were lower in groups treated with estrogen or with estrogen combined with progestogens when compared with those treated with TIB or placebo. These could be explained by mere chance, since this difference has no statistical significance. However, the TIB and CRT groups results were too disperse, with a higher SD, compromising the analysis of the data. The authors suspect that estrogen decreases those metalloproteases mRNA expression and significant differences were not seen because small sample size, treatment duration or therapy doses.

With respect to HPSE mRNA expression, the only parameter that showed differences between groups, the maximum values were observed in the TIB, EB and EB/MPA groups, although with no statistical significance in multiple comparisons. Apparently, the progestogens dydrogesterone and norethisterone acetate reduced the effect attributable to estrogen. This is in line with findings that this progestogen can reduce some estrogen effects.^12^

Surprisingly, the immunohistochemistry results did not replicate this significant finding for HPSE. A possible explanation for this apparent discrepancy between RNA levels encoding the enzyme and the actual level of protein in the vascular extracellular matrix could be a simultaneous increase in degradation of the RNA encoding this protein, or in the degradation of the actual protein, suggesting a greater turnover. This fact was also observed in a previous publication by our group.^11^

In contrast to an earlier publication, we did not observe in the present study an increase in the expression of MMP-2 or of MMP-9 with estrogen therapy, and this could be due to estradiol degradation impacted by portal hepatic circulation.^11^

However, it is important to highlight that the present study did not evaluate the degradation of the involved extracellular matrix proteins or of nucleic acids. On the other hand, the intention of including TIMP-2 was to know whether it would increase following the eventual increase of MMP-2. This would justify the lack of immunohistochemical alteration for tissue expression; however, the authors were not able to observe an increase in MMP-2, neither in TIMP-2.

In line with our findings, some studies have not found an increase in serum levels of MMP-9 in women treated with estrogen; however, in these studies, metalloproteinase was not analyzed in tissue, but only in plasma.^13^ ^14^ On the other hand, there is evidence of an increase in serum levels of MMP-9 in women with coronary arterial disease who received estrogen.^15^ It is worth noting that, in this study, conjugated equine estrogens were administered orally,^15^ while in the Christodoulakos GE et al study, the estrogen used was estradiol,^13^ and Wakatsuki et al^14^ administered conjugated equine estrogens orally or estradiol transdermally.

Marfella et al^16^ found lower levels of MMP-9 in atherosclerotic plaques in women who use hormone replacement therapy than in the plaques of women who have never undergone this treatment. However, it should be pointed out that these authors studied atherosclerotic plaques directly, while we have evaluated arteries with no apparent disease.

Sophonsritsuk et al^17^ reported that oral estradiol reduces MMP-9 expression in carotid arteries of female monkeys, irrespective of the postmenopausal period, although some inflammation markers had lower expression in monkeys in early menopause when compared with late menopause. Lekontseva et al^18^ reported a reduction in MMP-2 levels in the mesenteric arteries of castrated rats who received estrogen when compared with those who were not treated.

We have not found any other study that compares as many progestogens as our does; however, in a study involving a cellular model, Hwang-Levine et al^19^ observed that medroxyprogesterone acetate tended to increase MMP-9 activity in the absence of estradiol while progesterone reduced it; however, the effects were the opposite when estradiol was present. Although we did not observe any statistically significant difference in our study, there is a suggestion that medroxyprogesterone acetate reduced the effect of estradiol on the expression of MMP-2, of MMP-9 and of perlecan in the immunohistochemistry assays. This should be the basis for new studies.

One of the limitations of the present study was the small sample size, which reduced the potential for detecting differences among the groups. Had the sample size been larger, it is possible that differences could have gained statistical significance. Also, degradation of extracellular matrix proteases was not measured, neither of the nucleic acids involved. Another relevant point is that the present study evaluated normal arteries, and it is possible that the steroid effects studied would have been different on arteries with atherosclerotic disease.

## Conclusion

In conclusion, we have found differences among the groups in the expression of HPSE mRNA, and the highest values were observed in the animals treated with TIB, EB, and EB/MPA. The effects of sex steroids on the expression of extracellular MMPs are complex, and are probably dependent on an intricate interaction with other factors, which explains the limited agreement among existing studies. Despite the present study not having revealed statistical significance in the expression of carotid matrix components among treatments with different progestogens, our findings suggest that differentiated effects according to the progestogens used can exist. This information is important, considering the possibility of identifying progestagen with greater potential for endothelial harm and worse cardiovascular outcome in women. Also, these results could contribute toward outlining new studies.
